# Resource-Efficient Sensor Data Management for Autonomous Systems Using Deep Reinforcement Learning

**DOI:** 10.3390/s19204410

**Published:** 2019-10-11

**Authors:** Seunghwan Jeong, Gwangpyo Yoo, Minjong Yoo, Ikjun Yeom, Honguk Woo

**Affiliations:** 1 Department of Software, Sungkyunkwan University, Suwon 16419, Korea; party1996@skku.edu (S.J.); ikjun@skku.edu (I.Y.); 2 Department of Mathematics, Sungkyunkwan University, Suwon 16419, Korea; necrocathy@skku.edu (G.Y.); mjyoo2@skku.edu (M.Y.)

**Keywords:** autonomous system, sensor network, real-time data, digital twin, reinforcement learning, action embedding

## Abstract

Hyperconnectivity via modern Internet of Things (IoT) technologies has recently driven us to envision “digital twin”, in which physical attributes are all embedded, and their latest updates are synchronized on digital spaces in a timely fashion. From the point of view of cyberphysical system (CPS) architectures, the goals of digital twin include providing common programming abstraction on the same level of databases, thereby facilitating seamless integration of real-world physical objects and digital assets at several different system layers. However, the inherent limitations of sampling and observing physical attributes often pose issues related to data uncertainty in practice. In this paper, we propose a learning-based data management scheme where the implementation is layered between sensors attached to physical attributes and domain-specific applications, thereby mitigating the data uncertainty between them. To do so, we present a sensor data management framework, namely D2WIN, which adopts reinforcement learning (RL) techniques to manage the data quality for CPS applications and autonomous systems. To deal with the scale issue incurred by many physical attributes and sensor streams when adopting RL, we propose an action embedding strategy that exploits their distance-based similarity in the physical space coordination. We introduce two embedding methods, i.e., a user-defined function and a generative model, for different conditions. Through experiments, we demonstrate that the D2WIN framework with the action embedding outperforms several known heuristics in terms of achievable data quality under certain resource restrictions. We also test the framework with an autonomous driving simulator, clearly showing its benefit. For example, with only 30% of updates selectively applied by the learned policy, the driving agent maintains its performance about 96.2%, as compared to the ideal condition with full updates.

## 1. Introduction

Adopting machine learning techniques for autonomous systems (e.g., self-driving cars, robots, and industrial machines) have become popular for improving system performance as well as extending automation scenarios to the next level in a wide range of business domains. The Internet of Things (IoT) is important for intelligent autonomous systems: IoT enables these systems to make full use of operating log data in near real-time as the input for inference models, which are trained either online or offline.

From the point of view of cyberphysical system (CPS) architectures [1], intelligent autonomous systems often share the same visionary approach toward “digital twin” [2,3,4], where the status of a physical process is continuously updated to the intelligence part of a system and control commands for the physical process are executed through a standard interface of the system’s proxy. In general, the term digital twin underlines perfect synchronization and presumes transparent interfaces and mechanisms, thereby concentrating on the beneficial illusion of the complete digitization of physical processes. For example, a network of sensors and controllers deployed in a physical environment can be modeled as a database that supports transaction mechanisms and SQL-based common programming interfaces [5]. This abstraction intends to hide the complex interconnection among data entities and other parts of a system. However, there exist resource restrictions on sensors, networks, and database systems, (e.g., spatio-temporal sampling limitation, latency of sensor data updates, and limited database scales), that might limit the achievable level of timely data synchronization, which can pose challenges on establishing the abstraction in practice.

**Figure 1:**
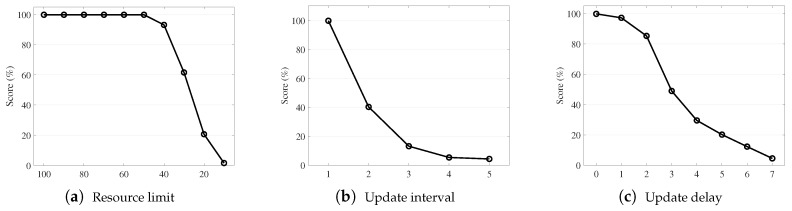
Effects of resource restriction: The driving test score of the simulator [6] with respect to (**a**) resource utilization limits for sensor data updates in percentages, where higher limits indicate more resource usages (on the X-axis); (**b**) average time interval in frames for consecutive updates of sensor data where large intervals indicate less resource usages; and (**c**) average time delay in frames for consecutive updates of sensor data where longer delays indicate less resource usage.

**Figure 2:**
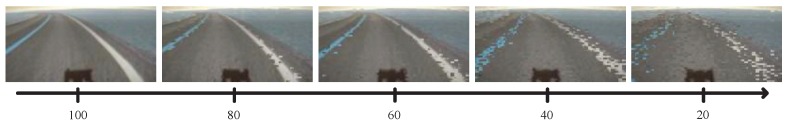
Effects of resource restriction: Observation examples in the case of Figure 1a where the numbers on the line denote the resource limits.

Figure 1 and Figure 2 illustrate the effects of constrained system resources in our example tests with the driving simulator [6]. Here, we focus on the test results only to briefly explain our motivation; we will describe the detailed test environment in Section 4. In Figure 1, the driving score on the Y-axis depicts the driving capability of the agent tested. The score was degraded as more resource constraints were specified for the sensing mechanism. On the X-axis, we set the restriction level imposed on resource usages as several synthetic stages based on configurable parameters such as (a) resource limits in percentages, (b) update time intervals in frames, and (c) update delays in frames. For example, the resource limits specify the portion of the newly updated parts on a scene image, emulating the constrained bandwidth availability for data transmission and processing of a network system with multiple narrow field-of-view sensors [7,8] or a multicamera sensing system [9,10]. This limitation was synthetically tailored for our case study with a driving simulator, as will be discussed in Section 4.4. Similarly, the update time intervals and update delays created different situations that emulate temporal limitations and imperfect data synchronization. As expected, having more restrictions caused the driving score level to significantly worsen. Figure 2 indicates the cause of degradation such that the driving agent made corrupted observations as a result of strict restrictions. These results motivated us to investigate a resource-efficient sensor data management strategy with the objective of enhancing the overall performance of a target application that relies on sensor data quality.

In this paper, we concentrate on the restricted resource issue of sensor data management for intelligent autonomous systems in which timely data synchronization is particularly important, but system resources for synchronization are restricted. To do so, we present the reinforcement learning (RL)-based data management framework D2WIN, which learns the scheduling policy of sensor data workloads under resource constraints and is located transparently between sensors and an application. This framework maintains the data quality and provides the application with robust interfaces to sensor data. Recent advance of RL combined with deep neural networks has increased the opportunity for automating and optimizing the system operation in many areas, e.g., cloud task scheduling [11,12], cluster resource management [13,14], network traffic scheduling [15], and database query processing [16]. These RL-based approaches commonly make use of repetitive operation patterns of a system under complex environmental conditions, thereby learning by experiences to make better decisions that meet the objective of system optimization [13]. In the same vein, D2WIN employs RL-based optimization techniques for managing sensor data, where job scheduling for updating data objects needs to adapt to the time-varying conditions of CPS applications. The main contributions of this paper are summarized below.

- We show how to adopt the real-time data model and RL techniques for autonomous systems by implementing the D2WIN framework (in Section 2).
- We address the RL scale issue in sensor data management by presenting the action embedding method LA3 (locality-aware action abstraction), which exploits distance-based similarities in sensor data and computes a large set of actions in a lightweight way, thereby efficiently dealing with the practical scenarios that use many sensor data objects (in Section 3).
- We show the competitive performance of the D2WIN framework compared to baseline algorithms under various experiment settings. We also apply the framework in a car driving simulator, and demonstrate that the driving agent yields stable performance (i.e., 96.2% in the driving score) under highly limited resource conditions such as 70% suppression of sensor updates (i.e., only 30% resource usage; Section 4).

## 2. Framework Architecture

**Figure 3:**
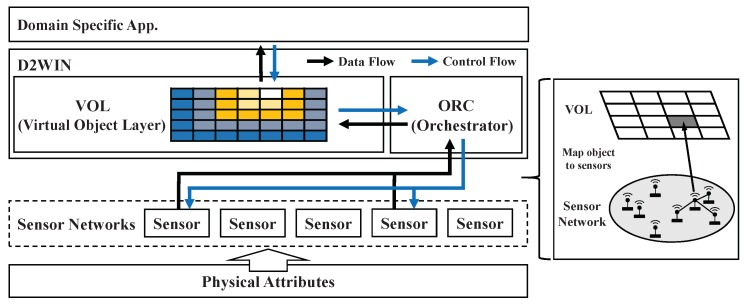
Conceptual system architecture for sensor data management: The D2WIN framework acts as a mediator between a domain-specific, data-intensive application, and a network system with multiple sensors.

In this section, we describe the overall system architecture of the D2WIN framework. As shown in Figure 3, the framework is layered between a domain-specific target application and sensor networks attached to physical attributes. It mediates interactions between theses especially when the workload generated by the application is data-intensive and time-constrained.

This framework consists of the virtual object layer (VOL) and the orchestrator (ORC). VOL provides the materialized view of a physical environment transparently for the application, and thus mitigates the uncertainty on data synchronization of the underlying sensor networks. ORC is responsible for scheduling the sensor data updates under resource constraints as well as maintaining the data quality on VOL to meet the application requirements. In this architecture, we assume that each data update is encapsulated as an individual job and updates are independent from each other in their execution paths. We employ deep RL techniques for developing a generic data orchestration mechanism for various autonomous systems, rather than concentrating on heuristics for specific conditions. It should be noted that our interest is not to develop another full-fledged data management system. Instead, we focus on the feasibility of a new feature, i.e., RL-based sensor data management, which can be integrated into a conventional data management system.

### 2.1. Virtual Object Layer for Sensor Data Management

As described in the overall architecture above, the virtual object layer (VOL) maintains a set of time-varying virtual objects, where the data value of each virtual object is continuously updated to be synchronized with its respective physical attribute. For each virtual object, *o*, in addition to its data value, o.value, we define a set of metadata. The staleness o.s∈N∪{0} denotes the time duration since the object was updated and synchronized, and the update processing time o.p∈N denotes the time duration needed for updating the object. In addition, the remaining processing time o.r∈N∪{0} indicates the residual time needed to complete the job of updating the object. In the context of real-time sensor data, we use a priori known approximation on the update processing time, o.p, similar to the authors of [17], and accordingly, we set $o.r=o.p+t_{sch}-t_{cur}$, where $t_{sch}$ and $t_{cur}$ are the latest scheduling time for updating *o* and the current system time, respectively. Furthermore, we assume the spatio-temporal locality of virtual objects, and present the index structure on $N^{2}$ for time-varying objects, e.g., $o_{3,4}$, thereby enabling us to map sensor data in a geographical–physical environment on the virtual object space. For simplicity, we use a two-dimensional index structure, but our proposed approach can also cover three-dimensional structures.

In our query semantics, we specify the temporal property of each query, *q*, with its staleness bound, q.b, and deadline constraint, q.d. Consistent with the locality of virtual objects, q.b is associated with individual objects, such that, e.g., $q.b_{i,j}=3$ indicates that evaluating *q* requires access to the virtual object $o_{i,j}$. Additionally, its staleness $o_{i,j}.s\leq 3$ should hold. Note that a query can be associated with more than one object, so it may have several staleness bounds. For evaluating *q* at a specific timestep, we say that the query *q* is satisfied at that time if $q.b_{i,j}\geq o_{i,j}.s$ holds for all $q.b_{i,j}$ and the time is within the deadline q.d. If there exists at least one satisfaction time within the deadline, the query is satisfied; otherwise, it is violated. These query semantics follow the query model in real-time databases [17,18]. Consequently, when the resource is restricted, the management objective of VOL is to maximize the rate of satisfied queries under resource constraints. It is also possible to consider such a case where the query satisfaction rate stipulates the data quality of VOL. In this case, the management objective of VOL is to optimize the resource usages under quality constraints. We use the term data quality, or quality of data (QoD), to represent such a query satisfaction rate.

### 2.2. RL-Based Data Orchestrator

**Figure 4:**
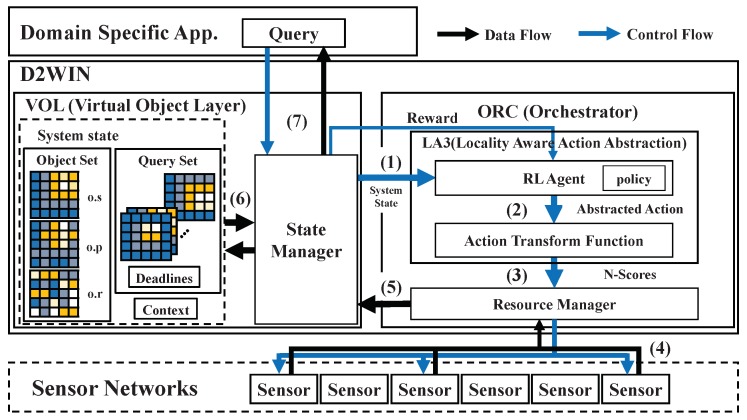
Framework architecture: The RL-driven multistage tasks (1–7) used to schedule the jobs for updating objects are described with the respective numbers on the control and data flows.

The orchestrator (ORC) is responsible for scheduling the jobs for updating objects, given specific constraints on resources or data quality, and it employs RL for achieving a stable scheduling policy across various operation conditions. To illustrate how ORC works, we describe its multistage tasks per timestep below. This is also shown in Figure 4, where orchestration control and sensor data flows are denoted by blue and black lines respectively. Notice that the timestep follows the time semantics of generic RL processing and corresponds to a unit time duration for system operation driven by an RL agent’s action [19].

(1) At each timestep, ORC retrieves the current information (system state) from the state manager of VOL as the input for the RL agent. The system state combines the representation of a set of queries, the metadata of virtual objects, and the context information. (2) The RL agent in ORC then initiates a scheduling decision for updating objects. Decision-making uses a two-step procedure to deal with the scale issue; this will be discussed later. (3–4) Given the scheduling decision, the resource manager in ORC launches the updating jobs on the sensor networks. (5–6) When a new set of sensor data is available, the state manager in VOL retrieves that set from the resource manager and applies it on the corresponding virtual objects. (7) The queries are then evaluated over the virtual objects, and their results are sent back to the application. Finally, the state manager updates the system state and prepares for the next timestep.

In the multistage tasks of the system flow per timestep, as explained above, VOL continuously shares its status with the RL agent in ORC through the encapsulated system state vector [qset;oset;ctx], where qset is the representation of the query set $\left\{\left({\left\{q_{1}.b\right\}}_{\forall i,j},q_{1}.d\right),\left({\left\{q_{2}.b\right\}}_{\forall i,j},q_{2}.d\right),\ldots \right\}$, oset is the representation of the virtual object set $\left\{\left(o_{1,1}.s,o_{1,1}.p,o_{1,1}.r\right),\left(o_{1,2}.s,o_{1,2}.p,o_{1,2}.r\right),\ldots \right\}$, and ctx is the context information. To allow for a wide diversity of modeling about physical attributes, we include the context information as an optional part in the system state. In general, we consider statistical properties of queries recognizable via a sliding window analysis as important features; these are included in the context information.

Algorithm 1 shows how the state manager in VOL works. Upon a set of sensor updates (finishedJobs in the code), the state manager renews the corresponding virtual objects’ value and staleness, and gets a set of new queries from the application. Then, the state manager evaluates all of the pending queries for the application and sends the reward feedback to ORC.

Note that the reward should be calculated properly for a given objective; the violated queries (or satisfied queries) are counted for presenting the negative feedback −Δ (or positive feedback +Δ), where Δ>0 is configurable. Given that system state [qset;oset;ctx] is continuously updated and shared as described, the RL agent in ORC can use its policy to drive actions and make scheduling decisions ((2) and (3) in Figure 4).

**Algorithm 1:** State manager process**1**oset,qset,ctx←initialize()   
// initialize the system state

**2**
**while**
notdone
**do**
**3**  finishedJobs←Orchestrator.execute(oset,qset,ctx)   
// (1)–(5) in Figure 4
**4**  **for**
$i,\;j\in \left[1,\ldots ,MaxScale_{x}\right]\times \left[1,\ldots ,MaxScale_{y}\right]$
**do**   
// (6) in Figure 4
**5**   $o_{i,j}.s\leftarrow \max\left(o_{i,j}.s+1,MaxStale\right)$**6**  **for**
w∈finishedJobs
**do****7**   $o_{w.index}.s\leftarrow 0$, $o_{w.index}.value\leftarrow w.value$    
// apply the result of job *w*
**8**  qset.add(Application.getNewQuery())**9**  reward←0**10**  **for**
q∈qset
**do****11**   satisfy←True**12**   **for**
$i,\;j\in \left[1,\ldots ,MaxScale_{x}\right]\times \left[1,\ldots ,MaxScale_{y}\right]$
**do****13**    **if**
$q.b_{i,j}>o_{i,j}.s$
**then****14**     satisfy←False;**15**   **if**
satisfy
**then****16**    reward←reward+Δ, qset.remove(q)**17**   **else****18**    q.d←q.d−1**19**    **if**
q.d<0
**then****20**     reward←reward−Δ, qset.remove(q)**21**  ctx←generateNewContext(qset)**22**  evaluateQuery(qset,oset)    
// (7) in Figure 4
**23**  feedback(reward)

## 3. Scheduling with Scalable Action Embedding

In this section, we present a two-step action embedding method that exploits the locality of sensor data to solve the RL scale problem for sensor-based autonomous systems. In D2WIN, each job for updating $o_{i,j}$ is associated with an individual transaction. In other words, a scheduling decision per timestep is represented as a set of object-wise transactions $\left\{w_{1},w_{2},\ldots \right\}$, where $w_{v}$ indicates that a job for updating the virtual object $o_{w_{v}.index}$ is scheduled. This action representation turns out to be combinatorial, i.e., O(Nk) for the case where the resource usage is constrained as no more than *k* updating jobs at each timestep (shortly, *k*-update constraint), and *N* is the size of the virtual object set. This type of direct translation by mapping object-wise transactions onto combinatorial action representation creates a problem related to large discrete action spaces in RL-based systems.

Specifically, with the aforementioned *k*-update constraint, the scheduling action for each timestep is illustrated to determine a subset of up to *k*-size from the *N*-size object set. If such combinatorial selection is translated to the action representation, the number of actions exponentially increases with large values of *k* and *N*. This poses a problem related to large discrete action spaces which has been described in RL-related research [20]. For instance, consider the case when we have k=51 and N=256 (i.e., 80% suppression, which allows a maximum of 20% simultaneous updating jobs in case of a $16^{2}$-scale VOL). In this case, there can be 25651≈1×1025 different discrete actions, which is far beyond what conventional RL algorithms are intended for.

### 3.1. Scalable Action Embedding for Sensor Data Management

To handle such Nk combination in the data management context, one might consider the score of objects and top-*k* ranking selection. In that case, the output of an RL network can be mapped on object-wise scores, where each virtual object is associated with a score in real number. Then, we can calculate the rankings of virtual objects using the scores, so this ranking-based scheme requires an *N*-parameterized output layer. This maps the RL action space from O(Nk) to an *N*-dimensional continuous action space; it remains difficult to train RL models for large scale system scenarios with many virtual objects in VOL.

**Figure 5:**
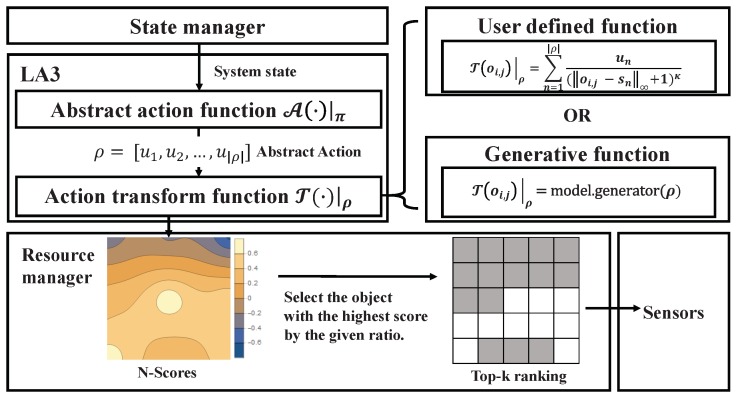
LA3 architecture: In the orchestrator (ORC), locality-aware action abstraction (LA3) treats the object-wise scheduling task as a two-step procedure (as illustrated in the left-box of diagram). The second step (${T\left(\cdot \right)|}_{\rho }$) is implemented based on either the user-defined function or the generative model-based function.

To solve this scale issue, we introduce the notion of a locality-aware action abstract method (LA3), which takes a two-step procedure, i.e., calculating the abstract action first and then the action transformation, so that the RL action space can be reduced to fit in a trainable range. More specifically, for each timestep, (a) the RL network produces the ρ-parametric abstract action as an output, and then (b) the action transform function based on ρ produces the score of *N* objects, where the number of parameters in ρ should be relatively small, i.e., |ρ|≪N. The LA3 method illustrated in Figure 5 follows this two-step procedure:

(1) $${\left(a\right)\;\;A\left(\cdot \right)|}_{\pi }\;\mapsto \;\rho ,\;\;\left(b\right)\;\;{\left\{T\left(\cdot \right){|}_{\rho }\right\}}_{\forall o\in oset}\;\mapsto \;N-\mathrm{scores}\;\mapsto \;\mathrm{Top}-k\;\mathrm{ranking}.$$

**Figure 6:**
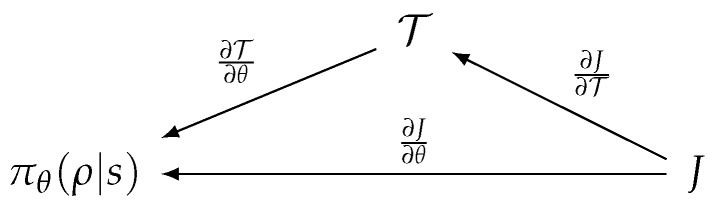
Branch diagram for the derivative of *J*.

Here, ${A\left(\cdot \right)|}_{\pi }$ is the abstract action function implemented by the RL policy π, and ${T\left(\cdot \right)|}_{\rho }$ is the transform function that converts an abstract action into the score of objects. As a consequence, the RL action space managed by the abstract action function can be scaled down while the ranking in the *N*-object set for performing the large scale object-wise scheduling is properly managed. When implementing this two-step action embedding method, it is important to have an appropriate action transform function $T\left(\cdot \right){|}_{\rho }$, which can be done by ensuring the properties below,

1. ${T\left(\cdot \right)|}_{\rho }$ is continuous on $R^{\left|\rho \right|}$. Based on this definition, for small ϵ>0, there exists δ>0 whenever $∥o-o^{\prime }∥<\delta$ implies $|T\left(o\right)-T\left(o^{\prime }\right)|<\epsilon$, with the given metric on the virtual object space ∥·∥. That is, nearby objects *o* and $o^{\prime }$ rank closely because the function reflects the locality of sensor data.
2. If we consider set *C*, the collection of local maximal points of $T\left(\cdot \right){|}_{\rho }$, and the dimension of virtual object space *d* (note that we assume *d* = 2 for simplicity), then we have $\left\{\rho \right|\;\lambda (T\left(c\right){|}_{\rho })\neq 0,\;\forall c\in C\}$ of measure zero [21] in the action space in $R^{\left|\rho \right|}$, where λ is the Lebesgue measure in $R^{d}$. It should be noted that object-wise selection by ranking is generally confined within the neighborhoods of the local maxima, and this property renders objects likely to be distinct in term of their ranking around the local maxima.
3. The derivative of $T\left(\cdot \right){|}_{\rho }$ must be bounded. This condition is required to keep the RL score function differentiable and for its derivative to be confined within a stable range. RL algorithms then work as if no transformation of actions are involved in learning. Figure 6 depicts the procedure used to compute the derivative of the score function *J* of the RL algorithm. $\pi_{\theta }\left(\rho |s\right)$ is a policy of the RL algorithm equipped with the weight θ, where *s* denotes the system state and ρ denotes the abstract action. $T:R^{\left|\rho \right|}\to \left(oset\to R\right)$ is a rewritten form of the action transform function, $T\left(\cdot \right){|}_{\rho }$, obtained by currying.

In addition, the runtime complexity of a transfer function should be restricted; this is because the operation environments of intelligent autonomous systems usually interact with physical processes in real-time. Thus we use a vectorization-based function implementation for ${T\left(\cdot \right)|}_{\rho }$ that satisfies the properties above. Suppose that we have a collection of fixed points, such as $S=\{s_{\left|\rho \right|}\in R^{2}|\;n\in \left\{1,2,\ldots ,\left|\rho \right|\right\}\}$, and a parameter set $\rho =\left[u_{1},u_{2},\ldots ,u_{\left|\rho \right|}\right]\in R^{\left|\rho \right|}$. Then, for all objects $o_{i,j}$, we have a user-defined function,

(2) $$T\left(o_{i,j}\right){|}_{\rho }\;:=\;\sum_{n=1}^{\left|\rho \right|}\frac{u_{n}}{{\left({∥o_{i,j}-{}_{n}∥}_{\infty }+1\right)}^{\kappa }},\;\;\mathrm{where}\;\kappa \in R^{+}$$

where κ is a correction constant denoting how far each term of the transform function can exert its influence, e.g., $\kappa =0.8+2(\sqrt{\frac{\left|\rho \right|}{N}})$. It is obvious that the function above is continuous. Each $u_{n}$ spans the function $T\left(\cdot \right){|}_{\rho }$, and each term of the summation has one local maximum except when $u_{n}$ = 0. The derivative is bounded by $\underset{\rho }{sup}{∥\rho ∥}_{\infty }$; i.e., the maximal value that the RL algorithm can produce.

### 3.2. Learning-Based Action Transformation

Here, we discuss how to generalize such an action transform function from the implementation perspective, particularly considering the restrictive environments where a stable function for action transformation can be rarely determined a priori but must be learned. If we regard the dimension of abstract action |ρ| as a hyperparameter of RL networks, we notice that large |ρ| settings generally make it difficult to achieve a stable policy (which corresponds to the abstraction action function). Indeed, there might be situations when the near optimal settings for |ρ| can only be found by intensive experimentation; this is the case because |ρ| has interdependency on both the action transform function and the number of objects *N* in our two-step structure of LA3. Therefore, it would be wise to acquire the latent dimension of a generative model through unsupervised learning [22].

**Figure 7:**
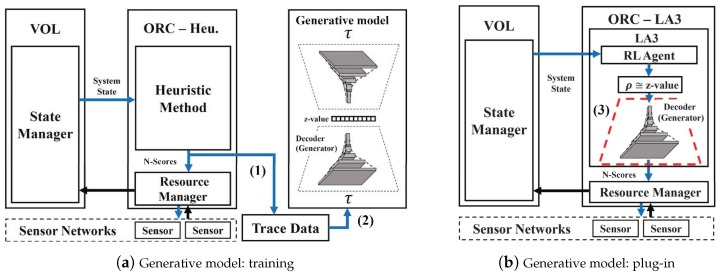
LA3 with the generative model: (**a**) The model is first trained to learn the features of *N*-scores from the trace data for relatively large *N*. Its decoder is then placed for LA3’s action transformation (**b**). As a result, the action space size of the RL agent becomes |ρ|≪N.

Below, we introduce a generative model-based action transformation. In doing so, we first consider an end-to-end policy based on a heuristic method that produces *N*-scores from the input state, and further suppose that the outputs of *N*-scores are assumed to be limited in their stability. Although the heuristic method is not competitive with our target, its samples can be used to learn how to extract the features of *N*-scores in VOL. Given the samples (τ∈TraceData in the code) collected by running, Algorithm 2 shows how to acquire a generative model to implement the action transform function: (1) Set the (non-RL) policy in ORC that produces *N*-scores using a heuristic method, e.g., stalest object first (SOF), query pattern in qset, or any user-defined function $T\left(\cdot \right){|}_{\rho }$ of which the input space is rarely confined on a manageable scale. (2) Train a generative model to extract the locality features from the latent space whose dimension is small enough to be learned. (3) Set the generative model as the action transform function of LA3. Figure 7a illustrates the example encoder–decoder structure of the generative model using a variational autoencoder (VAE). Figure 7b presents how to plug-in the decoder as the action transform function. Note that the z-value of the generative model is identified to be equivalent to ρ.

**Algorithm 2:**LA3 generalization process**1**env,Model←initialize()**2**policy←env.Orchestrator    
// (1) use ORC’s heuristic method, e.g., SOF
**3**TraceData←*∅*
**4**
**while**
|TraceData|<
SampleSize
**do**
**5**  τ←N-Scores←policy(env.state)**6**  TraceData.add(τ)**7**  env.step(*N*-Scores)**8**Model.fit(TraceData)    
// (2) train the generative model

**9**
$T\left(\cdot \right){|}_{\rho }\leftarrow Model.generator$

// (3)
$T\left(o_{i,j}\right){|}_{\rho }=\mathrm{Generator}\left(\rho \right)\left[i\right]\left[j\right]$


## 4. Evaluation

In this section, we evaluate our proposed framework D2WIN by comparing LA3 with several known heuristics across various experimental conditions. We first describe our implementation, and then show the experiment result with synthetic workloads, concentrating on the effect of LA3 that deals with the large-scale object-wise scheduling issue. We also explain our case study with the Donkey simulator [6] to clarify the transparent integration and performance benefit of the framework for sensor-based autonomous applications with specific resource constraints. For these experiments, we used a set of systems with an Intel CPU I7 8700 processor, 32GB RAM, and NVIDIA GTX 1060 3GB GPU. The ORC implementation in the framework is based on Tensorflow [23].

### 4.1. RL Implementation

Table 1 lists the models that we evaluated. Each model has its own scheduling policy that governs the updating jobs in VOL. To establish baselines for comparison, we implemented several heuristics-based (non-RL) models. The stalest object first (SOF) denotes the object-wise priority-based selection policy where the staleness determines the priority for each object. The earliest deadline first (EDF) is another model using the object-wise priority-based selection policy. EDF determines each object’s priority using the pending queries; a shorter remaining time to the query deadline creates a higher priority. In addition to the object-wise heuristics, we introduce another baseline that partially exploits the locality in VOL. Without any learning, the random action (RA) translates randomly generated |ρ|-numbers into a couple of object clusters using Equation (2).

Our proposed learning model LA3 is RL-driven, and it includes the action transform function. We employ multichannel representation for the system state; e.g., each query is represented on a single channel where the same grid structure of the virtual objects is given. We concatenate all system entities: oset,qset,ctx, as defined in Section 2.2. The concatenated state is used as the input for the RL agent. For LA3, we use the user-defined action transformation defined in Equation (2). We will show the other case with the generative model later.

Table 2 lists the hyperparameter value setting for training the RL policy . As an observation state of RL, we use the concatenated system state mentioned above. The RL agent produces the abstract action ρ as a parameter of the action transform function. Additionally, we use two RL algorithms, i.e., the soft actor-critic (SAC) [24] and the proximal policy optimization (PPO) [25]. We initially used PPO, considering the structure of our two-step action embedding procedure; however, when incorporating the action embedding in RL processing, there inherently exists a training difficulty that small weight updates might have significant effects on the RL policy. PPO employs trust region updating, so turning out to render the difficulty weakened. We also tested SAC, and experimentally verified its stable performance. PPO runs in parallel with multiple environments; it is fast, but often requires large computational resources beyond our machine capabilities for large-scale training scenarios. In comparison, SAC generally runs with relatively low resource usage, and so we can use it regardless of the scales in our scenario range. Note that we tested several RL algorithms, but PPO and SAC showed stable performances in most cases. For instance, with the deep deterministic policy gradient (DDPG) algorithm, we experienced training challenges that seem to be related with the extreme brittleness and hyperparameter sensitivity [26].

### 4.2. Environment Implementation

For evaluations under various environmental conditions, we define the configurable parameters in Table 3. Each parameter is specifically set within its value, as explained in the table, according to the experiment goal. Unless otherwise noted, the default value is used. The VOL scale (*N*) denotes the number of virtual objects. For evaluating the scalability of our proposed framework, we set the VOL scales differently from $16^{2}$ to $32^{2}$. We also set the upper bound of staleness MaxStale = 10; therefore, all o.s∈{0,…,10}. The resource (utilization) limit emulates the resource restriction on the jobs used for updating objects, which is defined based on the expected processing time (denoted as $E(o_{w}.p)$) as

(3) $$\mathrm{Resource}\;\mathrm{limit}\;\left(\%\right)=\frac{\left|\mathrm{Resbuf}\right|}{N\times E(o_{w}.p)}\times 100$$

Here, |Resbuf| denotes the resource capacity, i.e., the upper bound of the number of updating jobs in processing. The resource limit represents the normalized resource restriction, in that if every object can be updated at each timestep (i.e., |Resbuf| = $N\times E(o_{w}.p)$ is given) and all the queries can be satisfied with the freshest objects (o.s=0), the resource limit is 100%. We also use another term, where the suppression rate = 100 − resource limit.

We define two parameters for configuring the query-driven workloads: query arrival rate and dynamics. The query arrival rate specifies the average number of application-level queries at each timestep, where the arrival follows a Poisson distribution. The query dynamics denotes the level of changes between consecutive queries, which is governed by the query spatial difference. For two queries, the spatial difference specifies how much they are different in terms of the staleness bound ($q.b_{i.j}$); e.g., given queries $q_{t}$ and $q_{t+1}$, it can be measured by

(4) $$\mathrm{Query}\;\mathrm{spatial}\;\mathrm{difference}\;\left(\%\right)=\frac{\left|\;{\mathrm{diff}}_{\alpha }\left(q_{t},q_{t+1}\right)\;\right|}{|\;\mathrm{loc}\left(q_{t}\right)\;|+|\;\mathrm{loc}\left(q_{t+1}\right)\;|}\times 100$$

where $\mathrm{loc}\left(q_{t}\right)=\left\{\left(i,j\right)|\;q_{t}.b_{i,j}\neq MaxStale\;\right\}$ and ${\mathrm{diff}}_{\alpha }\left(q_{t},q_{t+1}\right)=\left\{\left(i,j\right)|\;\left|q_{t}.b_{i,j}-q_{t+1}.b_{i,j}\right|\geq \alpha \right\}$. Note that α∈{1,…,MaxStale} is the similarity threshold, and we set its default as α=1.

For handling multiple queries requested from applications, we implement a queue in the state manager in VOL, where pending queries in the queue are moved to available query slots in order of their priority, i.e., deadline. Note that the queries on query slots are visible as part of input state for the RL processing of ORC. Similarly, for handling multiple jobs for updating data objects, we implement a FIFO queue in the resource manager in ORC, where jobs are allocated on sensor networks as scheduled. At each timestep, ORC evaluates the importance (rankings) of objects, and then a set of jobs for updating objects in high ranks are queued.

### 4.3. Model Evaluation

**Figure 8:**
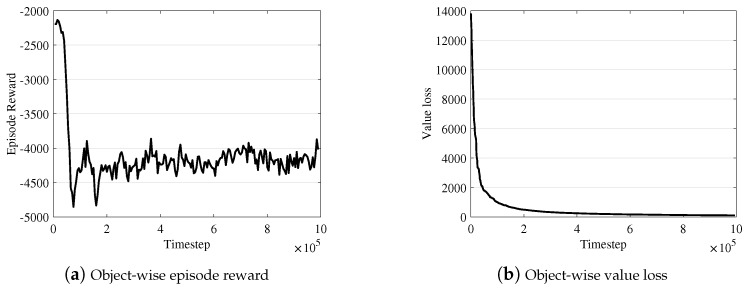
Object-wise reinforcement learning (RL) curve over time.

**Figure 9:**
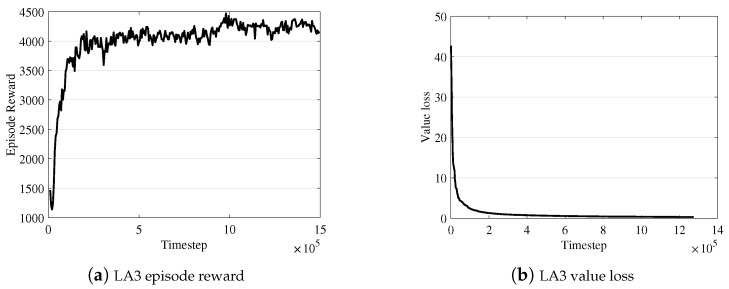
LA3 learning curve over time.

Comparison with object-wise RL. We intensively tested several RL algorithms to learn the object-wise selection policy, but we experienced many difficulties incurred by the large discrete action space. In our implementation, the naive objective-wise RL evaluates each object, o∈oset, as an individual action without any action embedding in RL processing. Figure 8 demonstrates the limitation of such a naive RL-based approach compared to Figure 9, which shows the learning curve of LA3. In the object-wise RL case, we observe that the loss in the learning curve converges (in panel (b)), but the reward does not well (in (a)). In the LA3 case, we observe that both the reward and the loss converge stably.

**Figure 10:**
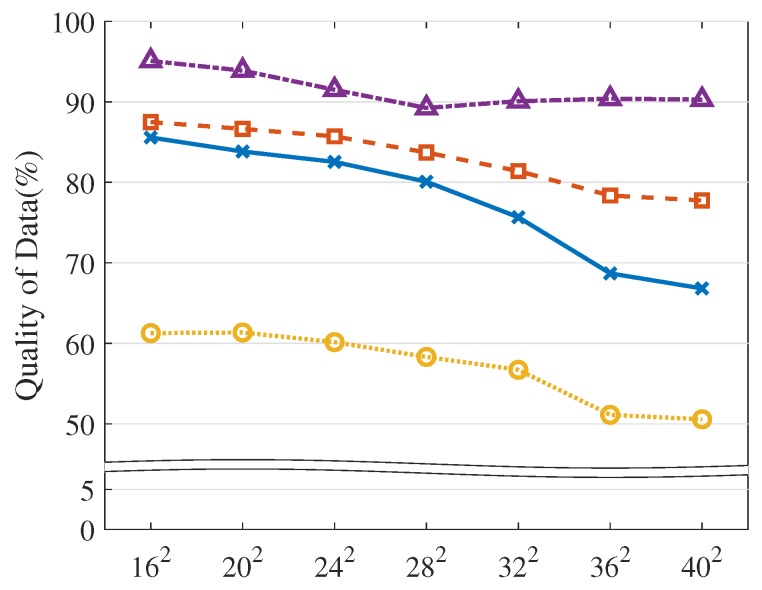
VOL scale.

**Figure 11:**
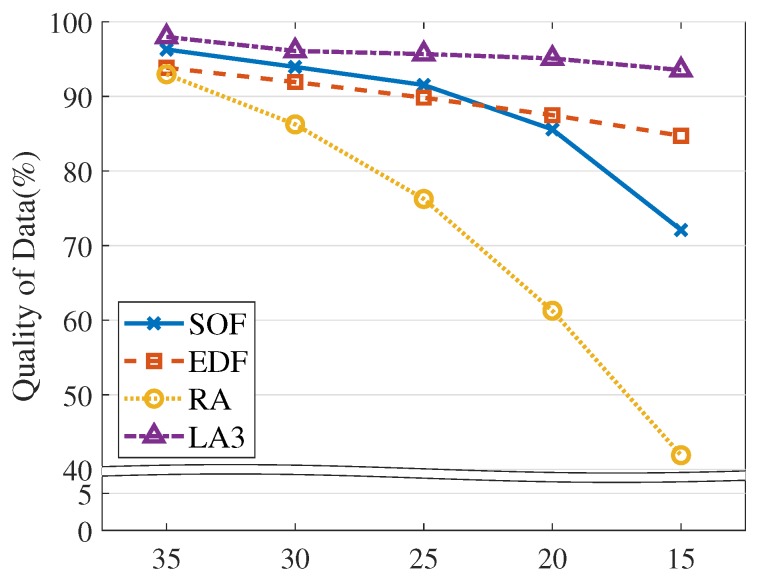
Resource limit.

**Figure 12:**
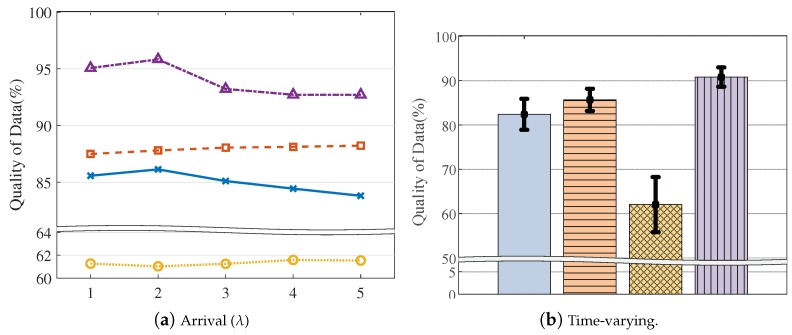
Query arrival time.

**Figure 13:**
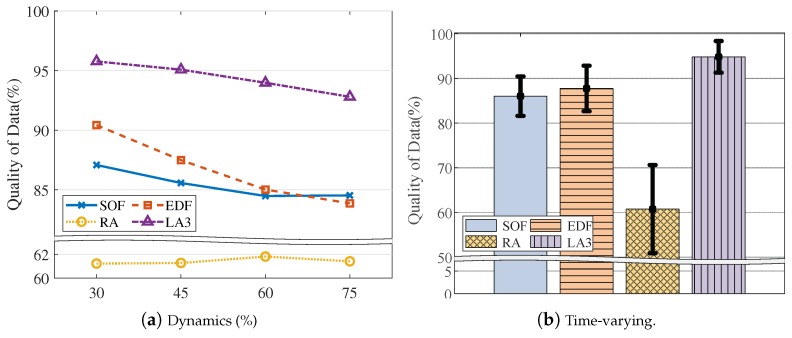
Query dynamics.

**Figure 14:**
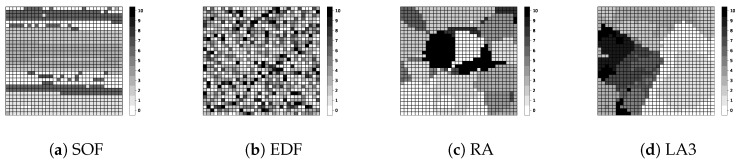
Object staleness snapshot examples by the models in Table 1.

**Figure 15:**
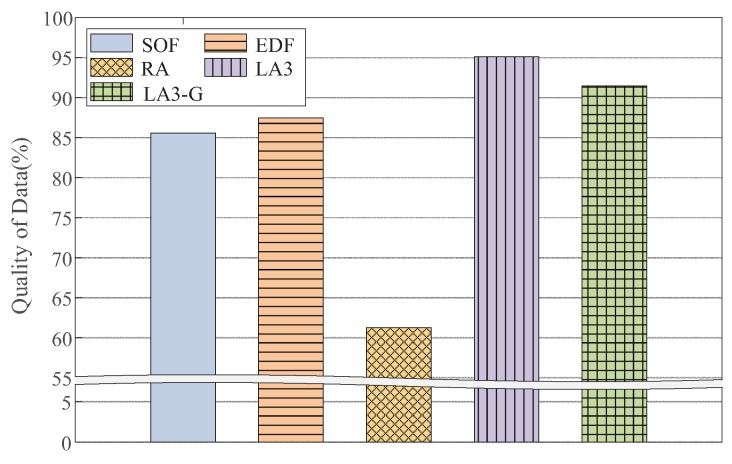
Performance of the generative model for action transformation: LA3-G and LA3 share the same structure for two-step action embedding, except that LA3-G exploits the generative model in Figure 7, but LA3 exploits the user-defined function.

Below, we explain the detailed experiment conditions and performance results. By default, we set the VOL scale as $16^{2}$ and the resource limit as 20%, as described in Table 3. Overall, LA3 outperforms the baseline heuristic methods, achieving higher QoD across the various experiment conditions, as shown in Figure 10, Figure 11, Figure 12, Figure 13, Figure 14 and Figure 15.

VOL scale. Here, we evaluate the performance of LA3 with respect to the VOL scales (on the X-axis). As previously discussed, the naive object-wise RL rarely revealed promising learning curves even for small scales; thus, we compare LA3 with the non-RL baselines, including SOF, EDF, and RA, in Table 1. Notice that the non-RL baselines are normally less sensitive to the scales, as they rely on either specific heuristics or randomness, which is different from RL-based approaches that should carefully deal with the scale issue (as explained by the case of the native object-wise RL).

As shown in Figure 10, LA3 outperforms the others for all of the scales. More precisely, it has yields that are 5.5∼12.5%, 9.1∼23.4%, and 30.9∼39.7% higher QoD than EDF, SOF, and RA, respectively. These results illustrate the advantage of LA3’s scalable action embedding. LA3 is able to enhance the object-wise scheduling scale by up to 40 times, as compared to the conventional RL-based operations. In this test, we set the dimension of abstract action |ρ|=16 for VOL scales $N=16^{2}$, $20^{2}$, and $24^{2}$, and |ρ|=25 for $N=28^{2}$, $32^{2}$, $36^{2}$, and $40^{2}$. Accordingly, we have $\frac{N}{\left|\rho \right|}=16∼64$.

Resource restriction. We also test the model robustness with respect to resource constraints. As shown in Figure 11, we configure the resource limit in Equation (3) at several stages (on the X-axis). In general, lower limits (lower resource usage) incur more suppression on updating jobs, which degrades QoD. It is observed that LA3 is robust at low resource limits; especially at the resource limit of 15%, LA3 shows higher QoD, up to 8.8% over EDF and 21.4% over SOF. Overall, LA3 shows 6.1% enhancement at average, compared with the highest heuristics EDF.

Query-driven workloads. In Figure 12 and Figure 13, we evaluate the model adaptability for different workloads that are configured by the query arrival rate (on the X-axis of Figure 12a) and the query dynamics (on the X-axis of Figure 13a). As observed, LA3 outperforms the others and shows stable performance even with intensive workloads. For example, in Figure 12a, the QoD achieved by LA3 is 4.5% higher than SOF, 8.9% higher than EDF, and 31.2% higher than RA when there are an average of five queries per timestep (i.e., 5 on the X-axis). Similarly, in Figure 13a, LA3 shows a higher QoD up to 8.3% over SOF, 8.3% over EDF, and 31.4% over RA for the case with 75% dynamics (i.e., 75 on the X-axis).

Furthermore, we also test the time-varying workloads. In Figure 12b, we set the query arrival rate to continuously change over time by the Poisson distribution $\lambda =1.5+W_{t}$, where $W_{t}$ follows Wiener process. Similarly, in Figure 13b, we set the query dynamics to continuously change over time among the predefined settings. In these figures, the error bar represents ±3σ intervals where σ is the standard deviation of overall QoD returns. In overall, LA3 yields the smallest variance, and this result underlines the scheduling adaptability of LA3 to dynamic workloads.

Figure 14 represents the different object staleness by the various models. In the figure, each example heatmap snapshots the objects’ staleness (all o.s∈oset in the system state) from VOL during a test operation; the darker areas indicate more stale objects and the lighter areas indicate less stale objects. It is observed that less stale objects are well clustered by RA and LA3 which use the same action transformation function in Equation (2). The snapshot examples in (c) and (d) reflect the locality-aware behaviors of the action transform function. Furthermore, LA3 learns to construct clusters that are likely to maximize the query satisfaction rate (i.e., QoD) for observable pending queries as well as for future queries that are not observed yet via RL processing. In comparison, RA generates random clusters without learning.

Generative model. So far, we have used the user-defined function for action transformation in LA3. Here, we use the generative model explained in Section 3.2. In Figure 15, by comparing LA3-G (with the generative model trained by the query pattern) with LA3, we demonstrate that the generative model itself performs competitively with LA3; it yields no more than 3.6% degradation. Additionally, both LA3 and LA3-G show better performance than the others. This result clarifies the feasibility of the LA3 action embedding for cases when a locality-aware user-defined function can be implemented and also when such a function must be learned based on experiences.

Test with state-of-the-art. Here, we test a state-of-the-art work that addressed an issue of large scale discrete action spaces in RL. As there have been rare few RL-based works in the literature of real-time and sensor data management, we implement a variant of the Wolpertinger algorithm [20], namely, WOL-M, and test it with our simulation environment (by replacing LA3 with the variant in Figure 3). Specifically, we employ a simple mapping from a group selection of nearby objects to a discrete action. This mapping confines the action space of WOL-M within a manageable size, i.e., 1 million, and enables us to adapt the Wolpertinger algorithm for our sensor data structure.

**Figure 16:**
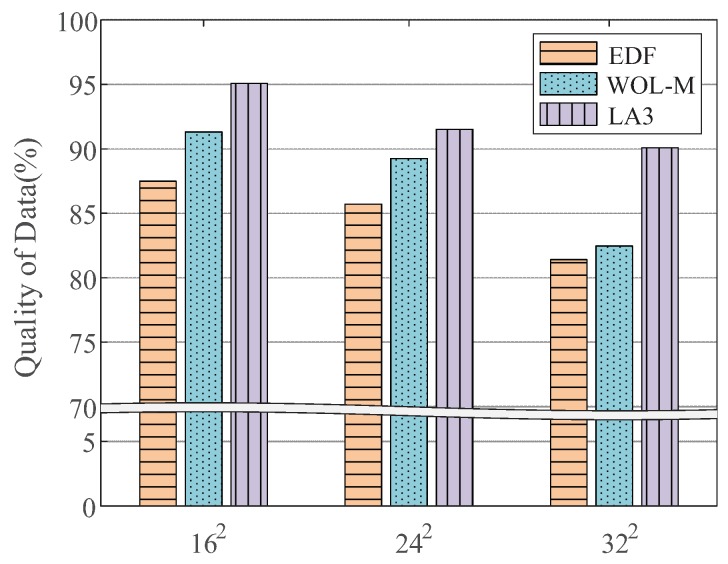
Performances test with a state-of-the-art variant.

Figure 16 shows the performance with respect to VOL scales (on the X-axis). We observe that for the case of $N=16^{2}$, $N=24^{2}$ and $N=32^{2}$, WOL-M yields up to 3.8%, 3.5%, and 1.1% higher QoD over EDF, respectively, whereas LA3 yields up to 7.6%, 5.8%, and 8.7% higher QoD. This result is in line with what we expected, in that the Wolpertinger algorithm has been tested up to the scale of 1 million discrete actions in [20]. As we focus on the a priori known locality of sensor data to address the scale issue and optimize LA3 action embedding using the locality, direct comparisons between our approach and the Wolpertinger algorithm are hardly meaningful. From the results, however, we rather confirm an advantage of using action embedding for large scale RL problems; both LA3 and WOL-M exploiting action embedding outperform EDF.

### 4.4. Case Study

In the following, we describe the case study with the Donkey simulator [6], which allows for conducting autonomous driving tests on randomly generated tracks. Through the case study, we demonstrate that the D2WIN framework is capable of maintaining the data quality on VOL for the driving simulation under a certain level of resource limitation. In the original operation, the full scene images in the game environment continuously provide raw input states for the driving agent. As shown in Figure 2 previously, when the images were partially corrupted due to some limitation, the driving agent can have serious difficulty in maintaining driving control. The framework addresses this data quality problem. Specifically, the framework enables the driving agent to operate successfully during driving tests under severe constraints, e.g., when 70% of updates are suppressed (in other words, a 30% resource limit is imposed). Note that all the driving tests here have been done without retraining the driving agent. This is consistent with the transparent integration style of the framework and a target application in real-world scenarios.

#### 4.4.1. Test System Implementation

**Figure 17:**
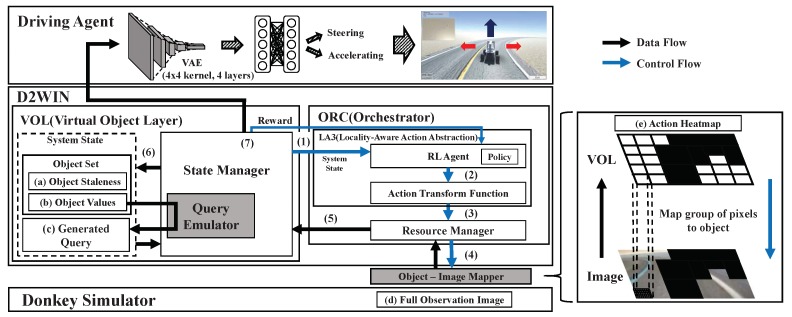
Case study system architecture: for this simulation test, we implemented two additional modules, i.e., query emulator and object-image mapper, in addition to the generic framework explained previously. The two modules are denoted by the gray box. Importantly, the implementation and integration of the two modules requires no modification in the simulator (which emulates a system environment with many sensors) or the driving agent (which emulates a domain specific application in Figure 4).

Figure 17 illustrates the overall system implementation, where the D2WIN framework acts as a mediator between the driving agent and the game environment (Donkey simulator), transparently providing the data layer that is able to control and transfer data updates from the environment to the driving agent application.

In addition to the generic integration structure of the framework, we implemented the object-image mapper by which a group of image pixels correspond to a virtual object in VOL, as if the image pixels within a single group were being sensed together by an individual sensor. This design is intended for emulating applications with multiple sensors. Although the Donkey simulator itself does not assume any details about the image sensing mechanism, we presumed that the car was equipped with multiple camera sensors [9].

**Figure 18:**
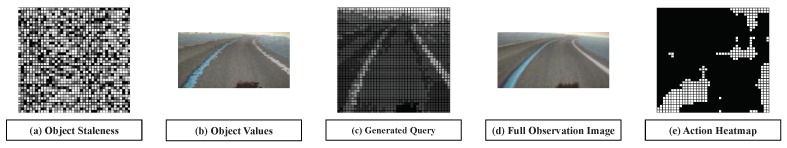
Internal data examples in the case study system: the examples (**a**–**c**) containing the system state are captured in the framework, example (**d**) is captured internally in the simulator, and example (**e**) is in the object-image mapper. Note that the full observation (**d**) is only available inside the simulator, and is not used by the framework or the driving agent; it is presented here only for comparison purposes.

At each timestep, ORC selectively schedules the updates for the virtual objects under specific resource constraints in the same manner as what was explained previously. The multistep procedure per timestep is described with the numbers in Figure 17. Furthermore, Figure 18 illustrates data that are internally captured at the different steps: (a) object staleness, (b) object values, and (c) staleness bound of a query in VOL, as well as (d) the snapshot image in the simulator. Additionally, ORC achieves the object ranking via LA3, which can be represented as the heatmap in (e), where the top-*k* objects turn into the action at this timestep.

Although an application normally generates queries, the simulator has no module implemented for making queries. Consequently, we implemented the query emulator inside VOL. This leverages the data values of the virtual objects at the previous timestep for query generation. More specifically, the query emulator first converts the data values (in partially corrupted images) managed by VOL into a grayscale representation, and then sets the staleness bound of a query by using the grayscale values, i.e., $q.b_{i,j}=\left\lfloor \frac{o_{i,j}.value}{255}*MaxStale\right\rfloor$. Then, for a query, *q*, the reward feedback is calculated based on the partial-query satisfaction rate, e.g., $\sum_{i,j}^{}\min\left(0,\;q.b_{i,j}-o_{i,j}.s\right)$. In the RL network, we add a convolution layer with a filter size equal to VOL scale so that the index structure can be maintained across the channels for the query and the objects. For the action transformation, we use the function defined in Equation (2). For this case study, we set the VOL parameters as shown in Table 4.

#### 4.4.2. Test System Evaluation

For evaluation of this case study system, we compare LA3 with non-RL heuristics, such as SOF and RA, which were previously used (in Table 1), as well as another baseline RO (random object). RO performs object-wise actions randomly at each timestep without any action embedding. This is different from RA in that it does not use any action transform function. Note that we set the deadline of all the internally generated queries q.d to 1, so EDF is excluded from the baselines.

**Figure 19:**
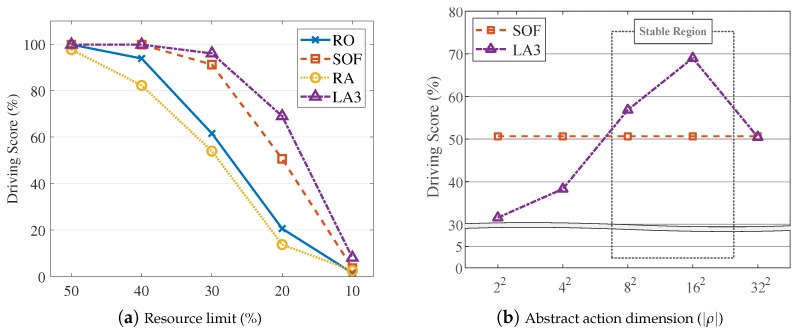
Performance evaluation: (**a**) we configure the resource limit in percentages at several stages (on the X-axis) and denote the driving score; (**b**) we configure the dimension of abstract actions |ρ| (on the X-axis). (**b**) For the VOL scale of $40^{2}$, we observe stable performance highly better than SOF with $\left|\rho \right|=8^{2},16^{2}$, but does not with $\left|\rho \right|=2^{2},4^{2},32^{2}$.

Resource restriction. We first test the Donkey simulator with D2WIN across various resource limits. Figure 19a shows the normalized driving scores with respect to the resource limits (X-axis). As shown, LA3 performs better than the others in most cases, showing 18.3% higher performance than SOF at a limit of 20%. More importantly, LA3 maintains 96.2% of the driving score even at a limit of 30%, as compared with the performance at a limit of 100%. This simulation result indicates that LA3 enables the driving agent to drive steadily in highly constrained driving environments, e.g., only 30% of sensor data updates are selectively applied in VOL and 70% are suppressed for bandwidth reduction.

In this simulation test, we use the RL-based driving agent that continuously takes the driving scene images as the input state to make decisions related to steering and acceleration. In the original simulation setting, we frequently observed that high-resolution inputs incurred delayed decisions, thereby lowering the score of the driving agent. However, the ORC of D2WIN manages inputs that consume fewer resources while still delivering good data quality to the driving agent. This enables the driving agent to acquire higher scores. It should be noted that we achieve this stability using RL-based data management without any sophisticated feature engineering or image processing.

**Figure 20:**
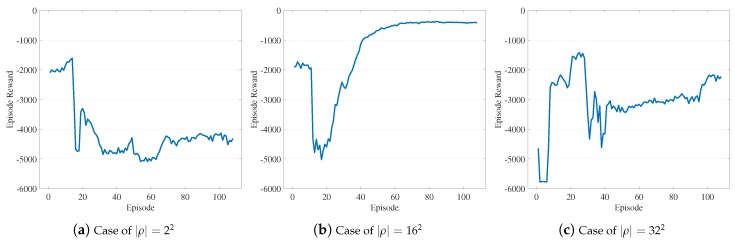
Comparison of stable and unstable cases: (**a**–**c**) the learning curves for the cases of $\left|\rho \right|=2^{2}$, $16^{2}$, and $32^{2}$, respectively, in Figure 19b, illustrating effects by |ρ| value settings, the dimension of abstract actions.

Dimension of abstract action. In the following, we analyze the effect of the dimension of abstract actions, denoted as |ρ|. To evaluate the effect only, we set the VOL scale to $40^{2}$, along with several |ρ| values. Figure 19b illustrates the dependency of LA3 on the dimension of abstract actions, where the X-axis denotes |ρ| values. We observe low performance with small |ρ| values due to their insufficient representational capacity, as compared to the VOL scale. As |ρ| increases, the performance is improved since a more representational capacity is achieved; however, at $\left|\rho \right|=32^{2}$, degradation is observed. This result is consistent with the issue related to large discrete action spaces of conventional RL algorithms, which was discussed in Section 3.1. It also demonstrates how LA3 efficiently addresses such a scale issue; the two-step action embedding of LA3 is able to deal with combinatorial actions of the $40^{2}$ VOL scale by exploiting RL algorithms that can deal with up to a $16^{2}$-dimensional action space. In line with this observed performance pattern, we also notice different learning curves in Figure 20 with respect to various abstract action dimension settings in Figure 19b.

Variant Objectives. Until now, our case study has demonstrated how the simulator utilized the virtual objects that are transparently managed by D2WIN, given the objective of maximizing the driving score (which is proportional to QoD according to the structure of internally generated queries) and the constraint of resource limits on sensor data updates. Here, we describe a variant scenario of the case study, which inversely defines its objective and constraint. In this test, we specify the resource utilization as the objective and QoD as the constraint, i.e., we want to minimize the number of sensor data updates while satisfying a given QoD requirement. To do this, we redesign the reward evaluating function as follows; $reward=\min(QoD_{t},\;Constraint)-ResUtil$, where $QoD_{t}$ is the QoD achieved at the current timestep, Constraint is the given QoD requirement, and ResUtil is the amount of resources that the RL agent can use.

**Figure 21:**
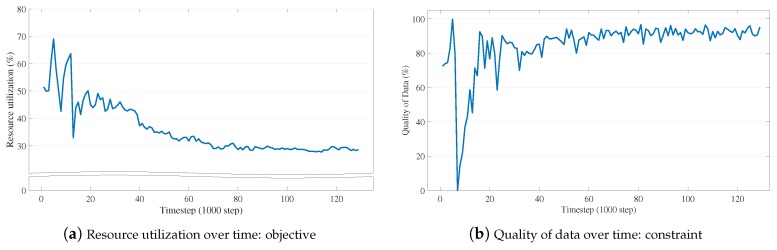
Variant objective test: LA3 for this test aims at minimizing the number of sensor data updates (so, reducing the resource utilization (**a**)) while satisfying a given QoD requirement (e.g., 95% (**b**)).

In Figure 21, LA3 shows its extensibility to this type of scenario. Given the 95% QoD requirement (Figure 21b), it continuously learns to reduce the resource utilization over time (Figure 21a). As a consequence, we achieve less than 30% resource utilization (more than 70% suppression) along with 95% QoD, which is consistent with the results of our previous tests.

## 5. Related Work

Different from conventional systems, research focusing on the area of real-time database systems has concentrated on time-related properties of data management such as temporal validity of data and timing constraints (or deadlines) of transactions [27]. Particularly, there have been few works investigating real-time data management for dynamic workloads, which exploit feedback control structures [17,18,28]. For instance, Kang et al. [17] proposed a feedback-based system architecture, in which the data freshness of temporal data and the miss ratio of deadline-constrained transactions can be controlled together through adaptive intervals for data updates. This approach can be viewed as conceptually compatible with modern intelligent system research directions, such as reinforcement learning (RL)-based system optimization in a dynamic environment; however, it has rarely been used to explore any learning-based scenarios.

Mnih et al. [29] considered Partially Observable Markov Decision Process (POMDP) for image classification. Specifically, they assumed a bandwidth limited observation and employed recurrent neural networks (RNNs) for sequentially extracting features from several different parts of a true image with attention. For sensor networks where the sensor nodes dynamically construct paths to a centralized database system to continuously update time-sensitive data, Elgabli et al. [30] proposed an asynchronous advantage actor-critic (A3C)-based scheduler to minimize the sum of the expected age of information (AoI) of sensors. They stipulated a small-scale constrained network, e.g., only a single node among 10 IoT sensor nodes can transmit its data at a time. Chowdhury et al. [31] recently introduced a drift adaptive deep RL-based scheduling and resource management strategy for handling heterogeneous IoT application requests. With resource-constrained IoT devices, the strategy takes into account responsiveness and energy consumption when scheduling dynamic requests with varying resource demands, especially dealing with sudden demand changes via adaptive learning. These RL-based system approaches share similar application conditions (i.e., limited bandwidth related to sensing for machine learning applications [29], single node updates at each time [30], and limited IoT resources [31]) with our work, in that workloads for sensor data updates should be suppressed due to a specific resource limit. However, our target applications relate to real-time data management for autonomous systems. Thus, the scale of RL action spaces is normally far beyond their concern.

There have been several works that have addressed the scale issue in the RL problem domain. Pazis et al. [32] proposed actions in a binary format, and showed how each action can be associated with a unique binary by learning the value function related with each of the digits. To deal with large discrete action spaces in RL, Dulac et al. [20] suggested a clustering-based action group that maps proto-actions onto a discrete set. Their work is relevant to our LA3, in that actions are calculated through more than just RL processing. However, in the context of sensor data management, we specifically leverage the locality of sensor data to implement the scalable action embedding strategy, and present two different action transformation methods, user-defined and generative model-based functions.

Recently several studies have presented RL-based car driving simulators and RL models that are trained with simulators [6,33,34,35,36,37,38,39,40]. RL algorithms as well as the variants based on deep neural networks, including A3C [35,41] Deterministic Policy Gradient (DPG) [36], Deep Deterministic Policy Gradient (DDPG) [37], and Deep Q-Networks (DQN) [38,39], have been exploited. Our case study utilizes the car driving simulation environment in the same sense as these studies. However, we rarely focus on learning a driving model; instead, we focus on achieving resource-efficient data management for the model with various resource-constrained environment conditions.

## 6. Conclusions

In this paper, we presented an RL-based resource-efficient sensor data management framework, D2WIN, which can be integrated with autonomous systems where the operation relies on sensor data streams. In doing so, we addressed the scale issue that occurs when adopting RL for sensor data management. This is done by exploiting the locality of sensor data as well as the two-step action embedding, LA3 (locality-aware action abstraction). We tested the framework with various workload conditions and also conducted a case study with a car driving simulator, demonstrating that D2WIN enables the driving agent to drive well even when the resources for sensor updates (i.e., for driving scene images) are severely limited.

Our future work will focus on extending the action transform function to cover diverse structures of physical environments by employing another learning model based on RL. We are also interested in working with various sensor-based autonomous applications, such as drones and robots, in future case studies.

## Figures and Tables

**Table 1:** Models in comparison.

Models	Description
Stalest Object First (SOF)	selection in the staleness order
Earliest Deadline First (EDF)	selection in the deadline order
Random Action (RA)	selection by random fixed point values
Locality-Aware Action Abstraction (LA3)	selection by RL agent with action transform function

**Table 2:** Hyperparameter description and value setting for training.

Hyperparameter	Description : Training RL (SAC and PPO)	Value	
		SAC	PPO
State	Object set, Query set, Context		
Action	Parameter ρ (Abstract action)		
Reward evaluation	Satisfied query : +Δ, Violated query : –Δ	Δ=1	
Network layer structure	MLP(2 Layer, [64, 64]) with layer normalisation		
Action transform function	The function converts an abstract action into ranking	User defined function (Equation (2))	
Discount factor (γ)	The constant for reflect future reward	0.99	
Learning rate (η)	The learning rate	0.0001	0.00025
Training frequency	The number of timesteps between updating the model	1	-
Gradient steps	The number of gradient descent steps in update	1	-
Batch size	The size of batch. In PPO, batch size is as follows: (number of agents) × (number of steps per update)	64	8 * 128
Replay buffer size	The size of replay buffer	50,000	-
Target smoothing coef.	The soft update coefficient	0.005	-
GAE parameter	The Generalized Advantage Estimator	-	0.95
VF coef.	The value function coefficient for loss calculation	-	0.5
Clipping estimator	The clipping range parameter	-	0.2
Hyperparameter	Description : Training generative model (VAE)	Value	
Network layer structure	VAE (3 convolution layers; filter size: 3 × 3; stride: 1; number of filters: [64, 32, 16])		
Learning rate (η)	The learning rate	0.00025	
Optimizer	The method of updating NN	Adam	
Latent dimension	The dimension of the output of the encoder in VAE	12	
Sample size	The number of data in the data set	50,000	
Epoch	The number of learning for the entire data set in NN	20	

**Table 3:** Virtual object layer (VOL) parameter setting.

	Description	Value (Default)
VOL scale	The number of virtual objects	$16^{2}$, $20^{2}$, $24^{2}$, $28^{2}$, $32^{2}$, $36^{2}$, $40^{2}$ ($16^{2}$)
	managed by VOL	
Resource limit	Rate of usable resources (%)	35, 30, 25, 20, 15 (20)
Query arrival rate	The average of query arrival in	1, 2, 3, 4, 5 (1)
	a Poisson distribution (λ)	
Query dynamics	The expected spatial difference	30, 45, 60, 75 (45)
	between two consecutive queries (%)	

**Table 4:** VOL parameter setting in the case study system.

	Description	Value (Default)
Resource limit	Rate of usable resources (%)	50, 40, 30, 20, 10 (20)
The size of ρ	The dimension of abstract actions	$2^{2}$, $4^{2}$, $8^{2}$, $16^{2}$, $32^{2}$ ($16^{2}$)
